# Pseudocoarctation of the Aorta: A Rare Incidental Finding During a Preoperative Assessment

**DOI:** 10.7759/cureus.65574

**Published:** 2024-07-28

**Authors:** Ahamed Shafeeq, Nandakumaran M, Prathapkumar G, Rajasekhar Ramesh, Amalan Christudhas

**Affiliations:** 1 Cardiology, Madras Medical College, Chennai, IND

**Keywords:** cardiac catheterization, cardiovascular surgery, interventional cardiology, ct aortogram, pseudocoarctation of aorta

## Abstract

Pseudocoarctation of the aorta (PCoA) is a rare congenital anomaly characterized by the abnormal kinking of the thoracic aorta. It is often incidentally diagnosed but gained clinical significance due to its propensity to develop aortic aneurysm and rupture. A standard diagnostic and treatment algorithm for PCoA is lacking, and also, the natural history of the disease is not well studied. We present here a case of PCoA with a fusiform aneurysm of the thoracic aorta to emphasize the need to differentiate it from true coarctation and to rule out associated complications.

## Introduction

Pseudocoarctation of the aorta (PCoA) refers to a kink or bend of the thoracic aorta at the level of the ligamentum arteriosum but not significant enough to cause obstruction to flow, as seen in true coarctation of the aorta. Embryonic theory of this abnormality is postulated to be due to the failure of compression of the third to the seventh segments of the aortic dorsal root and the fourth segment of the aortic arch [1]. The clinical manifestations are often nonspecific or may even present with complications as their first manifestation. One such case of pseudocoarctation that was diagnosed during a preoperative evaluation is elaborated on here.

This case was previously presented as an eposter at the 2023 Tamilnadu Cardiological Society Annual Conference on September 24, 2023.

## Case presentation

**Figure 1:**
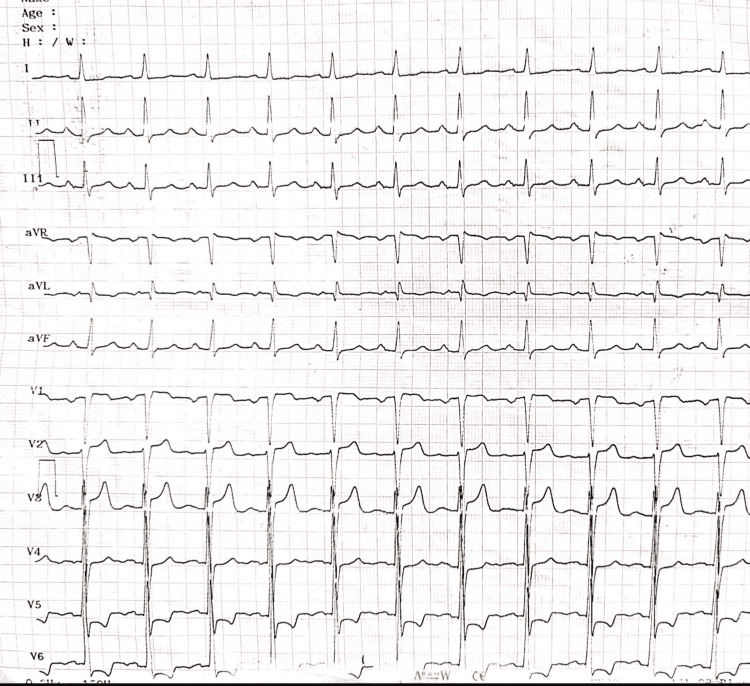
Electrocardiogram Electrocardiogram showing left ventricle hypertrophy with a strain pattern.

A 50-year-old male with a history of hypertension treated with enalapril (5mg/day) presented with dyspnea on exertion for two years. On clinical examination, blood pressure (BP) was 140/100mmHg with normal distal pulses in all four limbs and no radio radial or radio femoral delay. The electrocardiogram (Figure 1) showed left ventricle (LV) hypertrophy with a strain pattern, and the chest X-ray was fairly normal.

**Figure 2:**
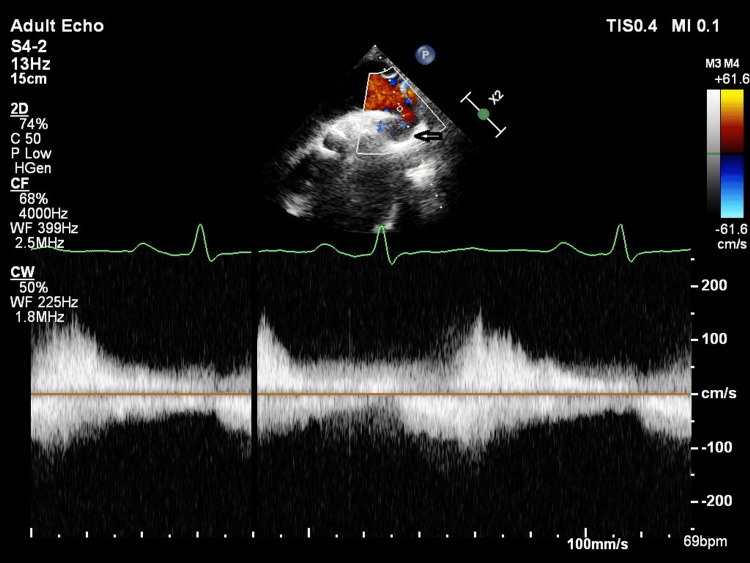
Transthoracic echocardiography Suprasternal view showing tapering of the descending thoracic aorta (arrow) and Doppler interrogation revealing no significant pressure gradient across the narrowing.

Transthoracic echocardiogram showed a tricuspid aortic valve with a dilated aortic root and severe calcific aortic stenosis (AS) and moderate aortic regurgitation, with mild LV systolic dysfunction (EF-48%). A suspected narrowing was visualised in the long-axis view of the descending thoracic aorta, and Doppler interrogation revealed the absence of any significant pressure gradient across the narrowing (Figure 2).

**Figure 3:**
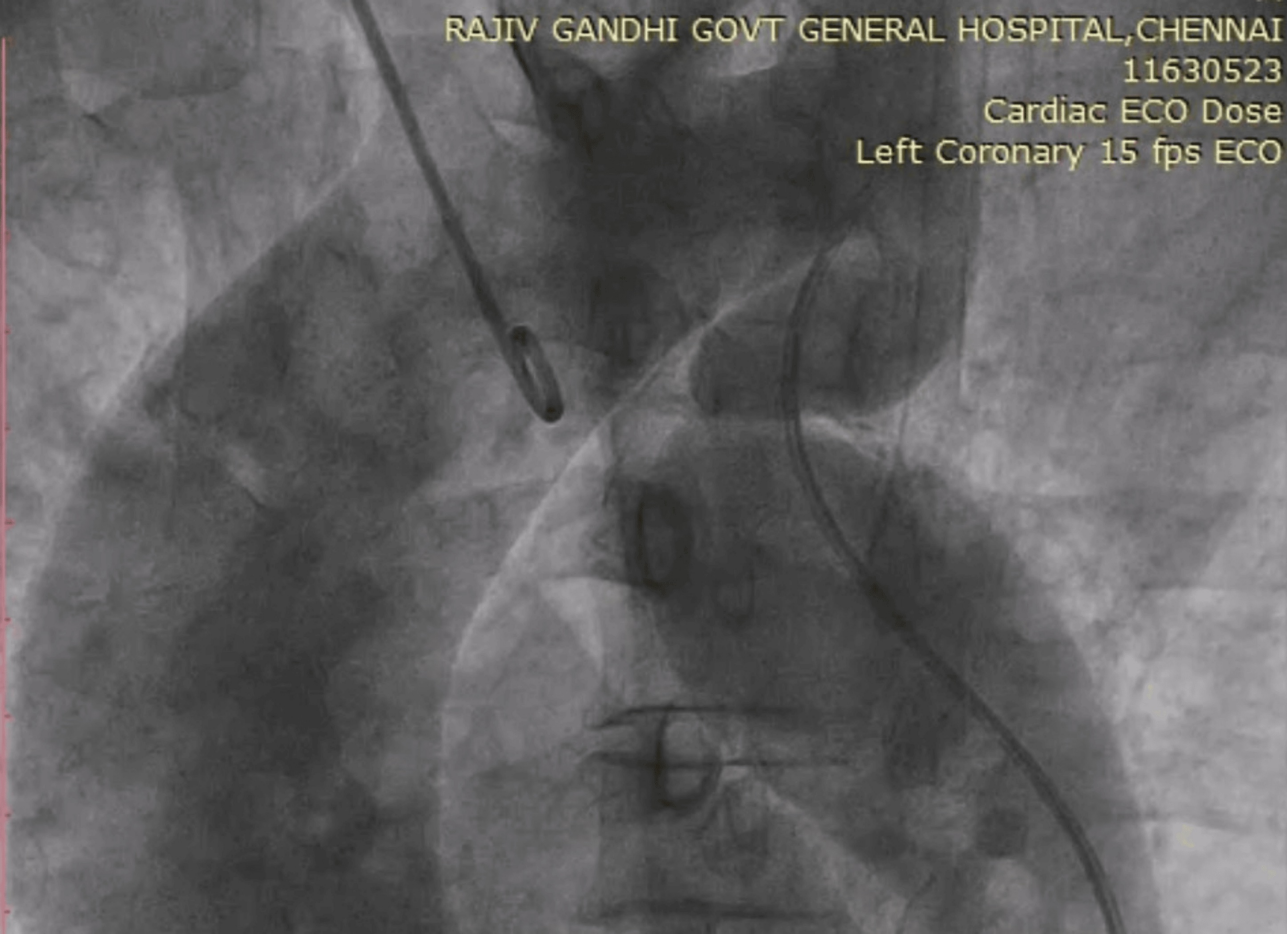
Aortogram Aortic root dilatation with kinking of the descending thoracic aorta and no collaterals were visualised.

In view of symptomatic severe AS and LV dysfunction, aortic valve replacement was planned and hence proceeded with a preoperative coronary angiogram (CAG). Through the femoral approach, 6F Judkins right catheter along with 0.014” guidewire failed to cross the aortic arch. Multiple attempts to cross the wire failed. The procedure was attempted through a radial approach with a 5F Tiger catheter and a successful CAG done, which showed normal coronaries. Subsequent angiography of the aorta with an automated power injector showed dilated aortic root with kinking of the descending thoracic aorta with no collateral circulation (Figure 3).

**Figure 4:**
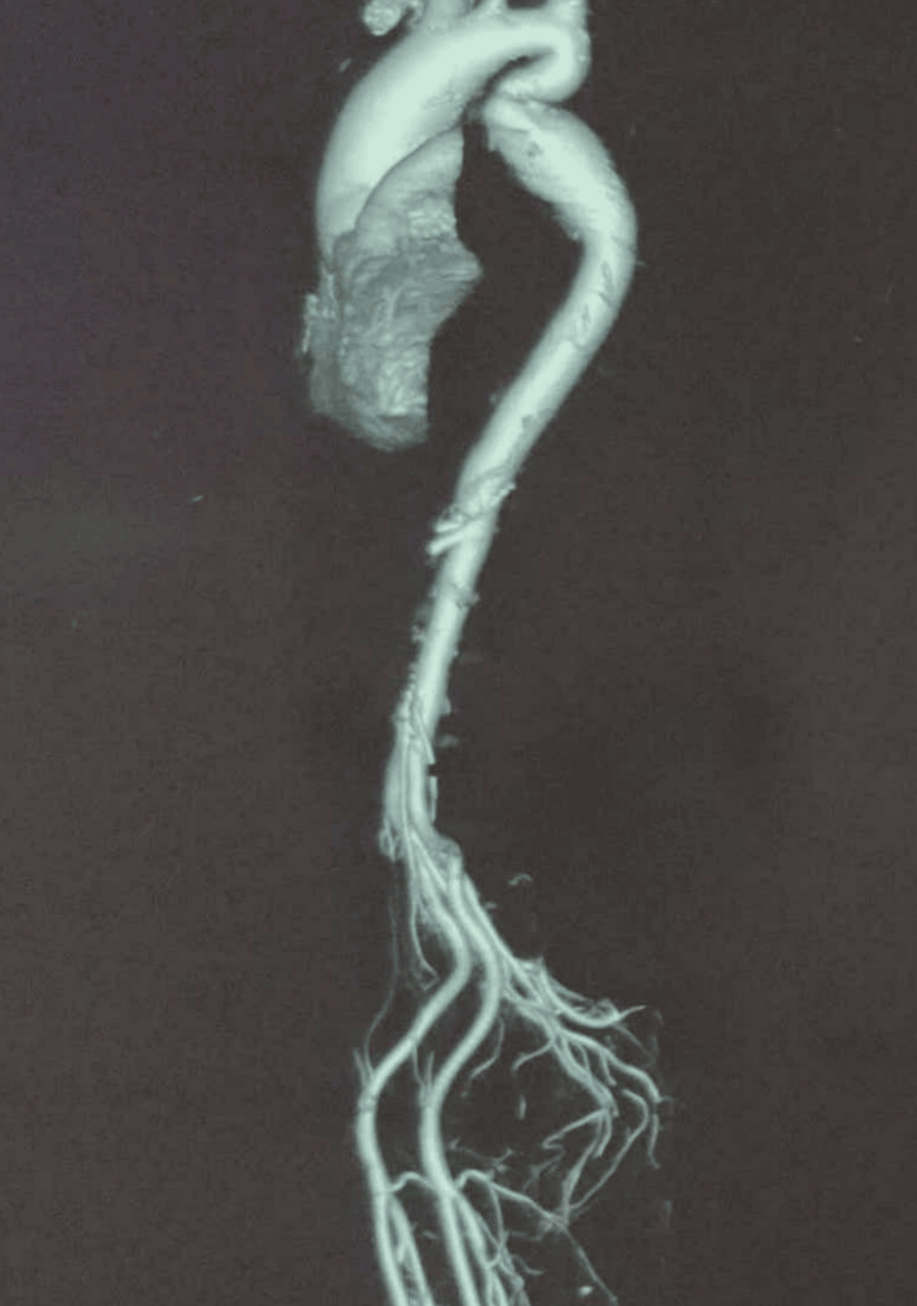
CT aortogram Fusiform aneurysm of aortic root with kinking of the descending thoracic aorta and absence of collateral circulation. CT: computed tomography

**Figure 5:**
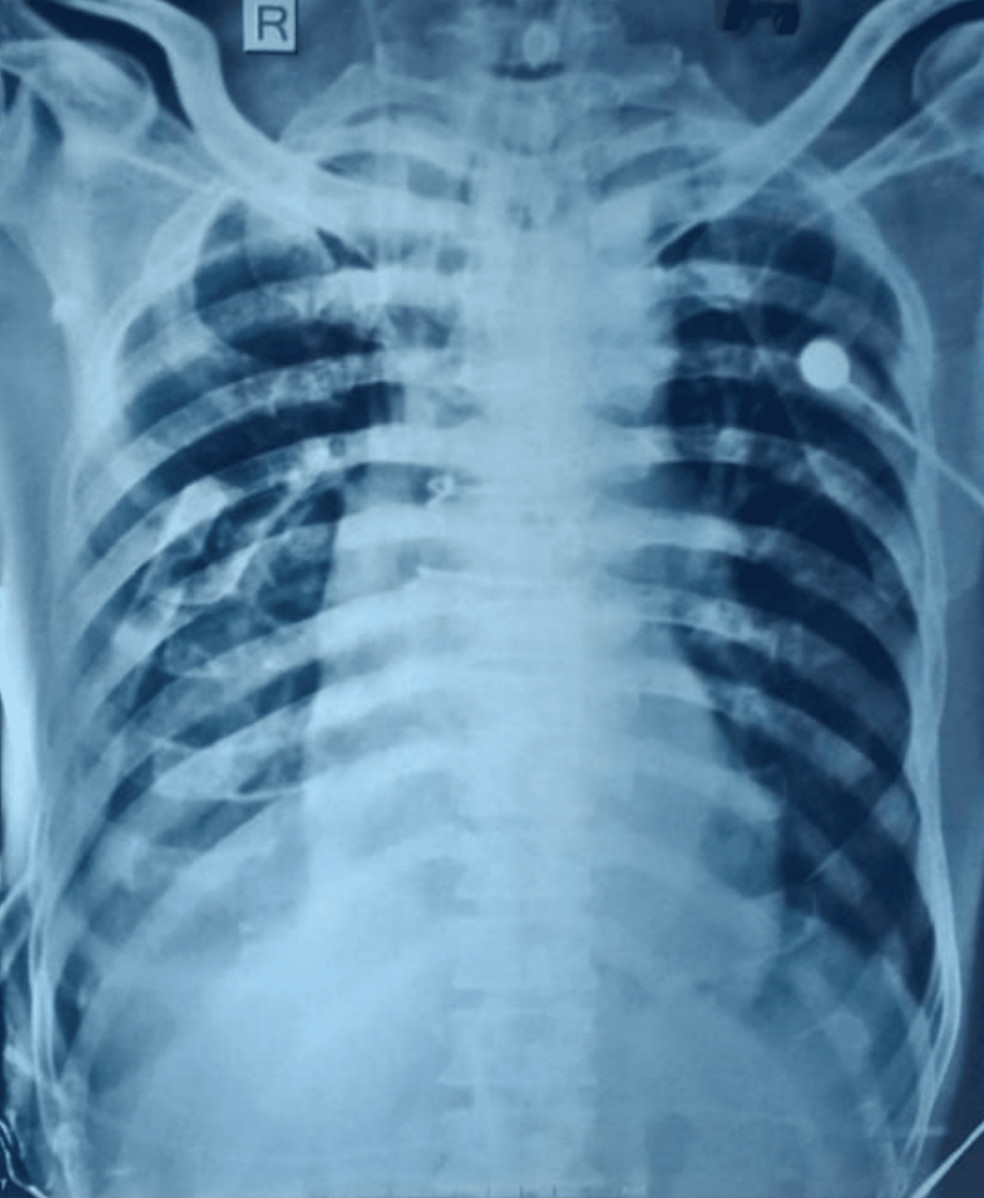
Chest X-ray Postoperative chest X-ray revealed haziness in the right lower zone suggestive of pneumonia.

Pressure data revealed no significant peak-to-peak gradient across the narrowed segment. Computed tomography (CT) aortogram revealed a fusiform aneurysm extending from the aortic root through the ascending aorta with a maximum dimension of 46mm, kinking of the descending thoracic aorta distal to the ligamentum arteriosum, and also an absence of collateral vessels (Figure 4). After discussing with the heart team, we proceeded with the surgery. Intraoperative findings revealed a calcified tricuspid aortic valve with pre- and post-stenotic dilatation across the narrowed segment. Aortic valve replacement was performed using a 21mm Sri Chitra TTK prosthetic valve, with a total cardiopulmonary bypass time of 178 minutes. The repair of the aortic root or pseudocoarctation was not attempted. The patient came off the bypass without difficulty. Postoperatively, the patient experienced a gradual drop in partial pressure of oxygen (pO2), accompanied by fever spikes. Chest X-ray revealed haziness in the right lower zone suggestive of pneumonia (Figure 5).

Cultures were taken, and antibiotics were escalated, with the decision being made to continue invasive ventilation. However, the patient could not be weaned off the ventilator and subsequently developed acute respiratory distress syndrome with superadded sepsis. Unfortunately, despite broad-spectrum antibiotic coverage and ventilator support, the patient succumbed two weeks post-procedure.

## Discussion

Pseudocoarctation is a rare development abnormality of the aorta consisting of an elongated or redundant aortic arch with kinking or buckling at the level of the ligamentum arteriosum. A diagnostic dilemma exists in differentiating hypoplastic aortic arch, pseudocoarctation, and coarctation, which are included in the same disease spectrum. Pseudocoarctation often has a benign clinical course and can be distinguished from true coarctation by the absence of significant blood pressure variation between upper and lower limbs and the absence of pressure gradient across the stenosis and without collateral circulation [1]. CT or magnetic resonance angiogram of the aorta is essential to evaluate the coarcted segment and associated aortic aneurysm or dissection and also the presence of collaterals [1]. Cardiac catheterization remains to be the gold standard for the measurement of pressure gradient [1].

In a systematic review published in 2014, 18 cases of pseudocoarctation were reported over 20 years, and it showed that the predominant symptom was hypertension with or without unequal BP in extremities, followed by dyspnea and dysphagia. The mean age of presentation was 43 years with no sex predilection [1]. Chest pain and back pain may be nonspecific or may be related to complications such as aneurysm, dissection, or rupture [2]. Few case reports of pseudocoarctation presenting as a superior mediastinal mass were reported [3]. Our case had hypertension without significant variation of BP in extremities; however, the exertional dyspnea may be attributed to the aortic valve pathology.

The most common association is aortic valve pathology such as unicuspid or bicuspid aortic valve and rarely variation in branching patterns of major arteries arising from the aortic arch [4,5]. Pseudocoarctation can be diagnosed in conjunction with chromosomal abnormalities, like Turner, Noonan, or Hurler syndrome, and congenital heart diseases, like a cervical aortic arch, ventricular septal defect, or patent ductus arteriosus [4]. Pseudocoarctation with polysplenia syndrome [6] and fused ectopic kidney were reported [7].

Surgical or endovascular techniques are reported in the management of pseudocoarctation [8]. However, the widely accepted approach is to wait and watch. The interventional approach is reserved for symptomatic patients and asymptomatic patients who develop a complication on follow-up. Tortuosity, thin aneurysmal wall, hypoplastic isthmus, or excess calcium deposits preclude an endovascular approach [9]. The 2022 American College of Cardiology/American Heart Association (ACC/AHA) guidelines on aortic aneurysm management recommend surgical intervention if the ascending aorta diameter is >55mm (Class 1) or >50mm if associated with high-risk features like coarctation (Class 2a) [10]. Still, no standard recommendations or guidelines are available to guide the timing and type of intervention, in managing pseudocoarctation.

## Conclusions

PCoA must be differentiated from true coarctation to accurately determine the nature of the pathology and decide on the appropriate management plan. This may help in avoiding unnecessary interventions, stress, and expenditure for the patient. Currently, only a few anecdotal evidences and case reports on pseudocoarctation are available in the literature. Future studies on the timing and modalities of treatment are needed. This case report, apart from emphasizing the need for accurate diagnosis of coarctation, also highlights the necessity for including its management algorithm in standard clinical practice guidelines.
